# Recognizing the unusual findings: Cases of desiderosmia

**DOI:** 10.1002/ccr3.2126

**Published:** 2019-04-02

**Authors:** Didar Yanardag Acik

**Affiliations:** ^1^ Division of Hematology Adana City Education and Research Hospital Adana Turkey

**Keywords:** desiderosmia, exhaust gasoline, iron deficiency anemia, menthol smell, pica

## Abstract

Iron deficiency anemia may cause a desire to smell. This has not been well defined by clinicians. In the cases we present, we have shown that there may be a desire to smell in iron deficiency anemia. We wanted to attract the attention of clinicians.

## 1 INTRODUCTION

Iron deficiency anemia (IDA) and pica are important health concerns worldwide. Pica is defined as the consumption of non‐nutritive substances that are inappropriate for an individual's development and cultural practices.^1^

Desiderosmia was defined by Hansen et al^2^ As the excessive desire to smell certain odors which develop along with iron deficiency, such as that observed in three cases of pica recorded in 2017. This condition was successfully treated with iron supplementation.

For conducting a research on IDA and pica, I assessed 522 patients and observed that three patients exhibited behaviors that are different from those of individuals with pica. They exhibited the desire to smell exhaust, gasoline, and menthol. Moreover, one of these patients expressed the desire to smoke in addition to smelling gasoline. I believed that these are interesting cases of pica. However, I encountered the work of Hansen et al during literature review and learned that these were considered as cases of desiderosmia a term that appropriately defined these cases. Therefore, I wanted to pay tribute to Hansen et al for their extremely helpful definition.

Here, I present these cases to further contribute to the literature.

## 2 CASE 1

A 43‐year‐old woman was diagnosed with IDA based on her blood test results. She had presented with weakness, fatigue, and palpitation. Because she exhibited the desire to smell exhaust and gasoline and to smoke, she was assessed for pica. The patient reported that she had previously been treated for iron deficiency, following which she had quit smoking and had no desire to smell exhaust and gasoline. The patient was treated with oral ferro fumarate 200 mg/d for 42 days. After the treatment period, her IDA was treated, and she quit smoking as well as lost the desire to smell exhaust and gasoline.

## 3 CASE 2

A 37‐year‐old woman was diagnosed with IDA based on her blood test results. She had presented with symptoms of anemia. On being assessed for pica, she reported that she had an excessive desire to smell menthol. The patient was treated with oral ferro fumarate 200 mg/d for 60 days. During her check‐up after the treatment period, her IDA was treated, and the desire to smell menthol was no longer present.

## 4 CASE 3

A 47‐year‐old woman was diagnosed with IDA based on her blood test results. She had presented with symptoms of anemia. On being assessed for pica, she reported that she had a desire to smell exhaust and gasoline. She was treated with intravenous ferric hydroxide sucrose 100 mg/d for 5 days. After 35 days of treatment, her IDA was treated based on her blood test results, and the desire to smell exhaust and gasoline was no longer present.

The clinical characteristics of the cases have been summarized in Table 1.

**Table 1:** Clinical summary of the cases

Patient	Gender	Age	Olfactory craving	Hb (g/dL)	Hct (%)	MCV (fL)	Ferritine (µg/L)	Treatment	Resolution of olfactory craving after iron therapy	Relapse of olfactory craving and pica
1	Female	43	Exhaust, gasoline odors	7.5	24.4	60.7	2.5	Ferro fumarate 200 mg/d, oral (42 d)	Yes (No desire to smoke)	No
2	Female	37	Menthol odor	9.9	32.9	74.1	9	Ferro fumarate 200 mg/d, oral (60 d)	Yes	No
3	Female	47	Exhaust, gasoline odors	8.9	32.1	70.1	5	Ferric hydroxide sucrose 100 mg/d, iv. (5 d)	Yes	No

Hb, hemoglobin; Hct, hematocrit; İV, intravenous; MCV, mean corpuscular volume.

## 5 DISCUSSION

Many articles were published in literature indicating that iron deficiency anemia causes overeating desire against certain substances or food. However, there is only one article of Hansen et al^2^ in literature comprised of three cases indicating that the iron deficiency anemia causing smelling desire. Ruvin Kumara and Wessling‐Resnick have researched the relationship between low iron condition of the body and behavioral scent functions by using the iron deficiency anemia model induced by diet in rats. As a result of this research, they have found that iron deficiency changes the smelling behavior.^3^ This study supports the fact that oddity in smelling behavior of our cases caused by iron deficiency.

I believe that many clinicians have encountered or will soon encounter cases of desiderosmia. Considering the current definition of this condition, I expect that clinicians would contribute further cases of desiderosmia to the literature, thereby enabling access to more interesting and illuminating information regarding this condition.

## CONFLICT OF INTEREST

None declared.

## AUTHOR CONTRIBUTION

Only one author contributed to this article.
